# Family and Peer Social Capital and Child Behavioral Outcomes in Japan

**DOI:** 10.3390/children11070840

**Published:** 2024-07-10

**Authors:** Jared M. Poff, Jonathan A. Jarvis, Mikaela J. Dufur, Shana L. Pribesh

**Affiliations:** 1 Department of Sociology, University of Utah, Salt Lake City, UT 84112, USA; 2 Department of Sociology, Brigham Young University, Provo, UT 84602, USA; jonathan_jarvis@byu.edu (J.A.J.); mikaela_dufur@byu.edu (M.J.D.); 3 Department of STEM Education and Professional Studies, Old Dominion University, Norfolk, VA 23529, USA; spribesh@odu.edu

**Keywords:** family social capital, peer social capital, child behavior, Japan

## Abstract

Background/Objectives: Child problem behaviors have been linked to immediate and long-term negative outcomes. Research has found that family and peer social capital have a strong influence on child behavioral outcomes. However, most research about social capital and child behavior problems has been conducted in Western contexts. Social capital may influence child behavior problems differently in non-Western sociocultural environments due to different family and peer dynamics. Methods: Using a sample from the Japan Household Panel Survey and Japan Child Panel Survey (*N* = 182), we expand this literature on various forms of social capital to the Japanese context with data that were collected between 2009 and 2014. We examine the relationship of family and peer social capital with children internalizing and externalizing problem behaviors using OLS linear regression. Results: Our results differ from what is commonly found in Western contexts. Whereas family and peer social capital are typically associated with both internalizing and externalizing problem behaviors in Western countries, we find that greater family social capital is associated with decreased externalizing problem behaviors but not internalizing problem behaviors in Japan, and peer social capital has no association on either type of problem behaviors. Conclusions: Our findings emphasize the importance of considering social and cultural contexts when exploring how social capital might encourage prosocial child outcomes.

## 1. Introduction

Child behavioral difficulties are important to assess due to the immediate and long-term negative impacts of such behaviors, including decreased cognitive development in the short term and problems related to physical health and employment in adulthood [1,2,3]. Important distinctions have been made between internalizing and externalizing child problem behaviors, with internalizing problems referring to a child’s inner emotional regulation and externalizing problems referring to their outward social behavior [4,5]. While internalizing problems are generally associated with issues of sociability and externalizing problems associated with poorer mental health, both types of problem behaviors can lead to a variety of unfavorable outcomes throughout childhood and adulthood [6,7]. 

One important factor that may affect the occurrence of child problem behaviors is social capital. James Coleman described social capital as the benefits, resources, and individual gains that come from social interaction [8]. Social capital can be derived from a variety of sources and has been examined specifically in both family and peer contexts. Family social capital includes familial relationships and investments in children that encourage child well-being and enable socialization [9]. Because the home is a primary source of social capital for children [10], family social capital has important implications for child behavioral outcomes [11]. Increased family social capital is associated with lasting positive effects on a child’s behavioral and intellectual development [12], including reducing the likelihood of deviant or delinquent behavior [9]. Peer social capital is a similarly important influence on child behavior and refers to the presence and nature of relationships that children have with peers [13]. Relationships with peers contribute to child and adolescent development, particularly at school [14], and are known to impact child well-being, life satisfaction, and risk behaviors [15].

While both peer and family social capital have generally been associated with positive child behavioral outcomes, social capital has been found to affect children differently across sociocultural environments [16]. For example, while greater family and school social capital are typically associated with favorable outcomes in Western studies, Jarvis and colleagues [17] found that some elements of social capital can exacerbate academic stress in South Korea. This suggests a need for more studies that examine child behavioral difficulties in non-western settings where unique cultural characteristics may impact associations between social capital and child behavioral outcomes. 

Japan provides a compelling setting to investigate child problem behaviors because of the intense family and school-related pressures that Japanese children and adolescents face. These pressures have been associated with violent behaviors and school avoidance [18], as well as decreased happiness and feelings of powerlessness [19]. Since home and school environments have been shown to affect child behavior in Japan in previous studies [18,20], it is expected that family and peer social capital impact child behavioral outcomes in Japan as well. However, to date, there has been little research that has examined how social capital affects the behavioral outcomes of Japanese children. Past Japanese research on child problem behaviors has found some general similarities with Western nations [21] but has less frequently included the effects of social capital. 

Recent studies in Japan on the topic (a) remain limited in their operationalization of social capital and rarely examine family and peer social capital concurrently, (b) often measure social capital from the caregiver’s point of view, and (c) do not present unified findings. For instance, Funakoshi and colleagues [22] find that individual and community-level parental social capital provides protective effects for children, while Yagi and colleagues [23] find no association between child behavior and social capital. Fujiwara and colleagues [24] find that caregiver cognitive and structural social capital is associated with decreased unhealthy behaviors in children. Takakura [25] measures cognitive and social capital as generalized social trust and finds that trust is negatively associated with drinking and smoking among youth. Finally, Nakano and colleagues [26] find that classmate social capital and family support can help reduce suicidal ideation in adolescents. 

While these studies are insightful, they do not clarify the relationships between family and peer social capital and child problem behaviors. There is some evidence that social capital in Japan may help reduce child internalizing and externalizing problem behaviors [25,26], but there is also evidence that social capital may not affect Japanese children in the same way as children in Western settings [23]. As such, further research is needed to examine peer and family social capital in Japan. 

Our research aims to (a) clarify the association between family and peer social capital and child behavioral outcomes in Japan, as well as (b) observe if family and peer social capital affect children differently in Japan than in Western settings. We hypothesize the following: (1) Similar to Western contexts [9] and in light of previous research in Japan [26], we expect greater family and peer social capital to be negatively associated with children internalizing problem behaviors; (2) Similar to Western contexts [15] and in light of previous research in Japan [25], we expect greater peer and family social capital to be negatively associated with children externalizing problem behaviors.

## 2. Materials and Methods

This study uses two datasets from Keio University’s Panel Data Research Center. The Japan Household Panel Survey (JHPS) is a nationally representative survey of households in Japan that began in 2009, and the Japan Child Panel Survey (JCPS) is a nationally representative companion survey beginning in 2010 that focuses on the children in JHPS households. The combination of these surveys allows for a joint examination of parents and children in Japan, as JHPS and JCPS participants have a “mainid” variable that allows parents and children to be connected. Keio University obtained consent from all subjects in the survey and managed all ethics committee approvals. Keio University makes the data available to scholars as secondary data available on their website (originally downloaded 22 November 2021; see “Data Availability Statement” for more details). 

Households were selected through two-stage stratified random sampling, and census survey districts were used as sampling units. Both surveys—which were conducted in Japanese and later translated into English—were administered by a surveyor who dropped off questionnaires to parents and children within the same household, and these surveys were then returned by mail or picked up by the surveyor. The response rates for included Waves are as follows: JHPS 2014 = 91.1%, JCPS 2012 = 57.5%, and JCPS 2014 = 45.6% (the response rate for JHPS 2009 is unfortunately not available from Keio University). We conducted OLS linear regression models to examine associations between family social capital, peer social capital, and other covariates of interest and child internalizing and externalizing problem behaviors. 

Table 1 reports descriptions for variables in our models. Our dependent variables are standardized scales of child internalizing and externalizing problem behaviors. Problem behaviors in the JCPS are assessed by parents through questions from the Strength and Difficulties Questionnaire (SDQ). SDQ questions have been commonly used to assess internalizing and externalizing problem behaviors in Western contexts [4], and the Japanese version of the SDQ has been found to have strong psychometric properties [21]. We follow standard practices to create scales for internalizing and externalizing problem behaviors [4], where the internalizing scale is created from the sum totals of two SDQ subscales (Peer Problems and Emotional Symptoms), and the externalizing scale is created from the sum totals of three SDQ subscales (Conduct Problems, Hyperactivity, and Prosocial Behavior). Each subscale contains five questions with three possible answer responses (“Not true”, “Somewhat true”, or “Certainly true”).

Peer Problems subscale questions include “Rather solitary, prefers to play alone”, “Has at least one good friend” (reverse coded), “Generally liked by other children” (reverse coded), “Picked on or bullied by other children”, and “Gets along better with adults than with other children”. Emotional Symptoms subscale questions include “Often complains of headaches, stomach-aches or sickness”, “Many worries or often seems worried”, “Often unhappy, depressed or tearful”, “Nervous or clingy in new situations, easily loses confidence”, and “Many fears, easily scared”. Conduct Problems subscale questions include “Often loses temper”, “Generally well behaved, usually does what adults request” (reverse coded), “Often fights with other children or bullies them”, “Often lies or cheats”, and “Steals from home, school or elsewhere”. Hyperactivity questions include “Restless, overactive, cannot stay still for long”, “Constantly fidgeting or squirming”, “Easily distracted, concentration wanders”, “Thinks things out before acting” (reverse coded), and “Good attention span, sees work through to the end” (reverse coded). Prosocial Behavior subscale questions include “Considerate of other people’s feelings” (reverse coded), “Shares readily with other children, for example, toys, treats, pencils” (reverse coded), “Helpful if someone is hurt, upset or feeling ill” (reverse coded), “Kind to younger children” (reverse coded), and “Often offers to help others (parents, teachers, other children)” (reverse coded). Both internalizing and externalizing problem behavior scales have good alpha reliability coefficients (α = 0.72, α = 0.78, respectively). Because the internalizing scale is a sum total of ten questions and the externalizing scale is a sum total of fifteen questions, the scales are standardized to facilitate proper comparison. 

Our main independent variables are peer and family social capital. Our scales for family and peer social capital are created from row totals of questions in the JCPS that ask children about their experiences in the past week. Family social capital questions include “I got on well with my parents”, “I felt fine at home”, and “I felt restricted by my parents” (reverse coded). Peer social capital questions include “I did things together with my friends”, “Other kids liked me”, and “I got along well with my friends”. Answer choices include “Never”, “Rarely”, “Sometimes”, “Most of the time”, and “Always”, and scales range in point total from 3–15. The alpha reliability coefficients for family (α = 0.71) and peer social capital (α = 0.72) are both good. 

We also include several covariates of interest. Child age is measured in the JCPS in years, with children in our sample ranging from ages 11 to 16. Child sex is assessed in the JCPS as male or female. Household income is measured continuously in the JHPS and ranges from 25–1500 (units of ten thousand yen). Mothers’ and fathers’ education are measured categorically in the JHPS with categories of “High school or less” (reference category), “Junior college or specialized school”, and “University or graduate school”. Finally, mothers’ employment is assessed in the JHPS and recorded as “Not working” or “Working”. Fathers’ employment was not included due to a lack of variance in responses, as nearly 100% of fathers reported being employed. 

We center our analysis on the year 2014, as this is the most recent wave available that allows for an analysis of our variables of interest while keeping a suitable sample size due to attrition occurring in later waves. While most variables in our analyses were measured in 2014, both family and peer social capital were measured in 2012 to capture their impact during the formation of 2014 child behavior outcomes. Additionally, because parental education was only assessed at the beginning of the JHPS, these measures are from 2009.

Our sample includes children who have available responses for 2009 measures of parental education, 2012 family and peer social capital questions, and 2014 measures of all other variables. This leaves our available analytic sample at 182 out of 187 children who had available responses in JCPS 2012 and JCPS 2014. We use multiple imputations to address missing data and keep our sample size at 182. Imputed variables include internalizing problem behaviors (1.7% missing), externalizing problem behaviors (1.7% missing), family social capital (19.8% missing), peer social capital (19.8% missing), household income (9.9% missing), fathers’ education (12.6% missing), and mothers’ education (13.2% missing). Variables that had no missing data and were registered as “regular” in the multiple imputation process include child age, child sex, and mothers’ employment. Due to the small sample size and the presence of heteroskedasticity, we use robust OLS linear regression. Finally, we used Stata 18 to perform our data analyses.

## 3. Results

Table 2 reports OLS linear regression results of our social capital and full models (Models 1 and 2, respectively) examining child internalizing and externalizing problem behaviors. Model 1 examines family and peer social capital alone, and Model 2 adds all other variables described in Table 1. In Model 1, we do not find significant associations between internalizing problem behaviors and either family or peer social capital, challenging our first hypothesis. However, we find that every unit increase in family social capital is associated with a 0.079 standard deviation decrease in externalizing problem behaviors (*p* < 0.05), though peer social capital remains nonsignificant. This gives partial support for our second hypothesis and suggests that while peer social capital may not play a significant role in the occurrence of child problem behaviors in Japan, greater family social capital can serve to decrease externalizing problem behaviors.

In Model 2, we add covariates of child age, child sex, household income, fathers’ education, mothers’ education, and mothers’ employment. For internalizing problem behaviors, we find that the relationship of family and peer social capital with behavior problems remains nonsignificant, again challenging our first hypothesis. However, similar to Model 1, we find that every unit increase in family social capital is associated with a 0.082 standard deviation decrease in externalizing problem behaviors (*p* < 0.05). Peer social capital is not associated with externalizing problem behaviors. Accordingly, even with the addition of control variables, greater family social capital is associated with decreases in child externalizing problem behaviors. This gives partial support to our second hypothesis and strengthens our findings from Model 1. 

## 4. Discussion

Our main finding is that greater family social capital is associated with decreased externalizing problem behaviors, even when controlling for differences in child characteristics such as age and sex. However, we do not find a similar association between family social capital and internalizing problem behaviors, and we do not find associations between peer social capital and either internalizing or externalizing problem behaviors. The reasons for this difference between family and peer social capital are unclear. Because externalizing problem behaviors concern outward social behavior, it may be that Japanese children look more to their family for learning proper social behavior than their peers. Furthermore, the influence of peer social capital may be weakened in Japan due to a cultural emphasis on social conformity [19], which may minimize differences in peer influence. However, our study does not identify specific mechanisms leading to this difference in the significance of peer and family social capital. 

Our findings contrast past research in Western settings, which has found that family and peer social capital affects both internalizing and externalizing problem behaviors [9,10,15]. However, our results exemplify how social capital can affect children differently across various sociocultural environments [16,17]. As such, there is a need for more research that examines family and peer social capital in settings that are less often examined. 

This study has a few limitations. For instance, while the JHPS and JCPS datasets allow for an examination of both child and parent data, our analytic sample is smaller than hoped for due to the dispersion of variables across the JHPS and JCPS (i.e., finding children that have the necessary individual and parent responses across relevant waves in two datasets). Additionally, while we are confident in our operationalization of family and social capital, these constructs differ from constructs like internalizing and externalizing problem behaviors in that they have no standard form of measurement. Accordingly, there is potential for studies to come to different conclusions regarding family and peer social capital based on differences in operationalization. This highlights the need for further work on family and peer social capital to help solidify our understanding of the concepts, particularly in settings where they have been less often examined. 

In conclusion, our results suggest that greater family social capital in Japan is associated with decreased child externalizing problem behaviors but not internalizing problem behaviors. On the other hand, peer social capital is nonsignificant for both types of behavioral outcomes. This suggests distinct patterns in how families and peers affect child behavior that may be specific to Japan. Further research is needed to (a) examine what mechanisms lead to this unique difference between family and peer social capital in Japan and (b) identify factors contributing to differences between Japan and comparable Western settings.

## Figures and Tables

**Table 1:** Description of variables.

Variable Name (Year Measured)	Variable Description	Mean/Proportion	Standard Deviation	Min– Max
Internalizing problem behaviors (2014)	Standardized scores from sum totals of two SDQ subscales; Peer Problems and Emotional Symptoms.	−0.01	1.09	−1.77–3.99
Externalizing problem behaviors (2014)	Standardized scores from sum totals of three SDQ subscales; Conduct Problems, Hyperactivity, and Prosocial Behavior.	−0.03	1.05	−2.06–3.49
Family social capital (2012)	Sum totals of three questions concerning family social capital	12.11	2.66	3–15
Peer social capital (2012)	Sum totals of three questions concerning peer social capital; point totals between 3–15	12.41	2.56	3–15
Childs’ age (2014)	Current years old	13.19	1.75	11–16
Childs’ sex (2014)	Male = 0 Female = 1	0.53 0.47	-	-
Household income (2014)	25–1500 (units of ten thousand yen)	521.58	212.21	25–1500
Fathers’ education (2009)	High school or less = 0 Junior college or specialized school = 1 University or graduate school = 2	0.45 0.18 0.37	-	-
Mothers’ education (2009)	High school or less = 0 Junior college or specialized school = 1 University or graduate school = 2	0.45 0.42 0.13	-	-
Mothers’ employment (2014)	Not working = 0 Working = 1	0.27 0.73	-	-
*N* = 182				

**Table 2:** OLS linear regression results of social capital and full models for standardized internalizing and externalizing problem behaviors.

	Model 1		Model 2	
	Internalizing	Externalizing	Internalizing	Externalizing
Family social capital	−0.033	−0.079 *	−0.040	−0.082 *
	[−0.096, 0.030]	[−0.151, −0.008]	[−0.106, 0.026]	[−0.151, −0.013]
Peer social capital	−0.038	−0.002	−0.033	−0.006
	[−0.100, 0.024]	[−0.078, 0.073]	[−0.096, 0.030]	[−0.078, 0.066]
Child age			−0.036	−0.165 ***
			[−0.115, 0.043]	[−0.252, −0.078]
Girl			0.347 *	−0.238
			[0.066, 0.627]	[−0.543, 0.066]
Household income			−0.001	−0.000
			[−0.001, 0.000]	[−0.001, 0.001]
Fathers’ education (rf: ≤ high school)				
Junior college or specialized school			−0.049	0.087
			[−0.461, 0.363]	[−0.389, 0.562]
University or graduate school			0.101	−0.157
			[−0.250, 0.453]	[−0.563, 0.249]
Mothers’ education (rf: ≤ high school)				
Junior college or specialized school			−0.188	0.182
			[−0.517, 0.142]	[−0.169, 0.534]
University or graduate school			−0.238	0.087
			[−0.771, 0.294]	[−0.446, 0.620]
Mothers’ employment			0.078	0.154
			[−0.232, 0.388]	[−0.188, 0.496]
Constant	0.685	0.882	1.299	3.175 ***
	[−0.140, 1.510]	[−0.104, 1.869]	[−0.071, 2.670]	[1.633, 4.716]
*N*	182	182	182	182

95% confidence intervals in brackets; * *p* < 0.05, *** *p* < 0.001.

## Data Availability

Restrictions apply to the availability of these data. Data were obtained from the Panel Data Research Center at Keio University and are available for download with the permission of the Data Management System of Panel Data Research Center at Keio University.

## References

[1] Jokela M., Ferrie J., Kivimäki M. (2009). Childhood Problem Behaviors and Death by Midlife: The British National Child Development Study. J. Am. Acad. Child Adolesc. Psychiatry.

[2] Narusyte J., Ropponen A., Alexanderson K., Svedberg P. (2017). Internalizing and externalizing problems in childhood and adolescence as predictors of work incapacity in young adulthood. Soc. Psychiatry Psychiatr. Epidemiol..

[3] Turney K., McLanahan S. (2015). The academic consequences of early childhood problem behaviors. Soc. Sci. Res..

[4] Goodman A., Lamping D.L., Ploubidis G.B. (2010). When to Use Broader Internalising and Externalising Subscales Instead of the Hypothesised Five Subscales on the Strengths and Difficulties Questionnaire (SDQ): Data from British Parents, Teachers and Children. J. Abnorm. Child Psychol..

[5] Mccarty C.A., Zimmerman F.J., Digiuseppe D.L., Christakis D.A. (2005). Parental Emotional Support and Subsequent Internalizing and Externalizing Problems Among Children. J. Dev. Behav. Pediatr..

[6] Owens J. (2016). Early Childhood Behavior Problems and the Gender Gap in Educational Attainment in the United States. Sociol. Educ..

[7] Willner C.J., Gatzke-Kopp L.M., Bray B.C. (2016). The dynamics of internalizing and externalizing comorbidity across the early school years. Dev. Psychopathol..

[8] Coleman J.S. (1994). Foundations of Social Theory.

[9] Dufur M.J., Hoffmann J.P., Braudt D.B., Parcel T.L., Spence K.R. (2015). Examining the Effects of Family and School Social Capital on Delinquent Behavior. Deviant Behav..

[10] Dufur M.J., Parcel T.L., Mckune B.A. (2008). Capital and Context: Using Social Capital at Home and at School to Predict Child Social Adjustment. J. Health Soc. Behav..

[11] Crosnoe R. (2004). Social Capital and the Interplay of Families and Schools. J. Marriage Fam..

[12] Dufur M.J., Parcel T.L., McKune B.A. (2013). Does Capital at Home Matter More Than Capital at School? The Case of Adolescent Alcohol and Marijuana Use. J. Drug Issues.

[13] Ream R.K., Rumberger R.W. (2008). Student Engagement, Peer Social Capital, and School Dropout Among Mexican American and Non-Latino White Students. Sociol. Educ..

[14] Bester G. (2019). Stress experienced by adolescents in school: The importance of personality and interpersonal relationships. J. Child. Adolesc. Ment. Health.

[15] Lau M., Li W. (2011). The extent of family and school social capital promoting positive subjective well-being among primary school children in Shenzhen, China. Child. Youth Serv. Rev..

[16] Drukker M., Buka S.L., Kaplan C., McKenzie K., Van Os J. (2005). Social capital and young adolescents’ perceived health in different sociocultural settings. Soc. Sci. Med..

[17] Jarvis J.A., Corbett A.W., Thorpe J.D., Dufur M.J. (2020). Too Much of a Good Thing: Social Capital and Academic Stress in South Korea. Soc. Sci..

[18] Kawanishi Y. (2009). Mental Health Challenges Facing Contemporary Japanese Society: The “Lonely People”.

[19] Houri D., Nam E.W., Choe E.H., Min L.Z., Matsumoto K. (2012). The Mental Health of Adolescent School Children: A Comparison among Japan, Korea, and China. Glob. Health Promot..

[20] Poff J.M., Jarvis J.A., Shafer K., Dufur M.J. (2023). Maternal and Paternal Psychological Distress and Child Behavior in Japan. J. Child Fam. Stud..

[21] Matsuishi T., Nagano M., Araki Y., Tanaka Y., Iwasaki M., Yamashita Y., Nagamitsu S., Iizuka C., Ohya T., Shibuya K. (2008). Scale Properties of the Japanese Version of the Strengths and Difficulties Questionnaire (SDQ): A Study of Infant and School Children in Community Samples. Brain Dev..

[22] Funakoshi Y., Xuan Z., Isumi A., Doi S., Ochi M., Fujiwara T. (2021). The Association of Community and Individual Parental Social Capital with Behavior Problems among Children in Japan: Results from A-CHILD Longitudinal Study. Soc. Psychiatry Psychiatr. Epidemiol..

[23] Yagi J., Fujiwara T., Yambe T., Okuyama M., Kawachi I., Sakai A. (2016). Does Social Capital Reduce Child Behavior Problems? Results from the Great East Japan Earthquake Follow-Up for Children Study. Soc. Psychiatry Psychiatr. Epidemiol..

[24] Fujiwara T., Takao S., Iwase T., Hamada J., Kawachi I. (2012). Does Caregiver’s Social Bonding Enhance the Health of their Children?:The Association between Social Capital and Child Behaviors. Acta Med. Okayama.

[25] Takakura M. (2011). Does social trust at school affect students’ smoking and drinking behavior in Japan?. Soc. Sci. Med..

[26] Nakano M., Yamazaki C., Teshirogi H., Kubo H., Ogawa Y., Kameo S., Inoue K., Koyama H. (2022). How Worries about Interpersonal Relationships, Academic Performance, Family Support, and Classmate Social Capital Influence Suicidal Ideation among Adolescents in Japan. Tohoku J. Exp. Med..

